# Bayesian Spatio-Temporal Analysis and Geospatial Risk Factors of Human Monocytic Ehrlichiosis

**DOI:** 10.1371/journal.pone.0100850

**Published:** 2014-07-03

**Authors:** Ram K. Raghavan, Daniel Neises, Douglas G. Goodin, Daniel A. Andresen, Roman R. Ganta

**Affiliations:** 1 Kansas State Veterinary Diagnostic Laboratory/Department of Diagnostic Medicine/Pathobiology, College of Veterinary Medicine, Kansas State University, Manhattan, Kansas, United States of America; 2 Bureau of Epidemiology and Public Health Informatics, Kansas Department of Health and Environment, Topeka, Kansas, United States of America; 3 Department of Geography, College of Arts and Sciences, Kansas State University, Manhattan, Kansas, United States of America; 4 Department of Computing and Information Sciences, College of Engineering, Kansas State University, Manhattan, Kansas, United States of America; 5 Department of Diagnostic Medicine/Pathobiology, Kansas State University, College of Veterinary Medicine, Manhattan, Kansas, United States of America; University of Minnesota, United States of America

## Abstract

Variations in spatio-temporal patterns of Human Monocytic Ehrlichiosis (HME) infection in the state of Kansas, USA were examined and the relationship between HME relative risk and various environmental, climatic and socio-economic variables were evaluated. HME data used in the study was reported to the Kansas Department of Health and Environment between years 2005–2012, and geospatial variables representing the physical environment [National Land cover/Land use, NASA Moderate Resolution Imaging Spectroradiometer (MODIS)], climate [NASA MODIS, Prediction of Worldwide Renewable Energy (POWER)], and socio-economic conditions (US Census Bureau) were derived from publicly available sources. Following univariate screening of candidate variables using logistic regressions, two Bayesian hierarchical models were fit; a partial spatio-temporal model with random effects and a spatio-temporal interaction term, and a second model that included additional covariate terms. The best fitting model revealed that spatio-temporal autocorrelation in Kansas increased steadily from 2005–2012, and identified poverty status, relative humidity, and an interactive factor, ‘diurnal temperature range x mixed forest area’ as significant county-level risk factors for HME. The identification of significant spatio-temporal pattern and new risk factors are important in the context of HME prevention, for future research in the areas of ecology and evolution of HME, and as well as climate change impacts on tick-borne diseases.

## Introduction

Human Monocytic Ehrlichiosis (HME) is a frequently reported tick-borne disease in the south central and southeastern USA. The severity of the disease ranges from a mild non-symptomatic infection to death in humans. Although most known HME cases are not fatal, approximately 3–5% of all ehrlichial infections in the USA result in deaths despite patients receiving appropriate care [1]. Prevention almost entirely relies upon how well humans can avoid ticks; vaccines are not available and managing ticks or quantifying management effectiveness can be difficult. *Ehrlichia chaffeensis*, an obligate intercellular bacterium affecting the monocytes and macrophages is responsible for HME, and infections are vectored by the Lone Star tick, *Amblyomma americanum*, a widespread tick species in the central Midwest and southern regions of the USA. A second bacterium *E. ewingii*, that target the granulocytes also results in similar clinical symptoms but is relatively less frequently encountered [2].

The number of reported cases and the spatial distribution of HME have steadily increased over the past decade since the Centers for Disease Control and Prevention, USA (CDC) designated HME as a reportable disease [2], and per Kansas Administrative Regulations, Ehrlichiosis was first reportable in Kansas in the year 2000. State epidemiologists believe during this period in Kansas, the focus area of this study, HME has gone from a disease that was reported only from a few counties in the southeast corner of the state to being more numerous and widespread across the eastern part of the state. Spatio-temporal dynamics of tick-borne diseases could be influenced by geospatial factors including climate, land cover/land use and socio-economic conditions, which are currently not known for HME in Kansas but could facilitate our understanding the mechanisms of HME transmission and guide disease control strategies under the current social, climatic and environmental changes.

Prior research on HME using GIS and remote-sensing methods has established some very useful hypotheses. Yabsley et al., (2005) [3], identified the relationship between *E. chaffeensis* and several geospatial variables (land cover/land use, forest fragmentation) using kriging surfaces and logistic regressions. Wimberly et al., (2008) [4] later showed the importance of including model parameters to account for spatial autocorrelation and spatial randomness in geostatistical studies using *E. chaffeensis* and *Anaplasma phagocytophilum* examples. This study also identified similar meteorological and land cover/land use variables as important predictors for *E. chaffensis*. The spatial distributions of the same pathogens were predicted using habitat-level ecological variables in the Mississippi valley by Manangan et al., (2007) [5] wherein the relative importance of variables (soil moisture and forest cover) at different spatial scales was shown. The former two studies considered relatively large regions for the spatial extent, and all had used seropositivity rates among deer populations as indicators of county or public land-level ehrlichial pathogen prevalence.

Influential factors affecting the prevalence and distribution of diseases could differ from one region to another due to natural changes in the geography, and they are quite scale-dependent [6], [7]. In addition, the relevance of certain spatial determinants often becomes evident only in the presence of others. Particularly, climatic variables are often confounded or interactive with other influential factors viz., land cover/land use and socio-economic characteristics and can be difficult to detect [8], [9]. Further, much of the prior work on HME spatial epidemiology is based on conventional statistical approaches that have limitations when epidemiologic datasets are analyzed. Bayesian hierarchical modeling has been recognized as a powerful analytical technique to provide more robust posterior estimates, since they allow for incorporating errors that may arise from mean or median estimates of the independent covariates and observed data through the use of prior probability distributions [10], [11].

The purpose of this study was to evaluate the presence of spatio-temporal autocorrelation and the relationship between key environmental, climatic and socio-economic factors with HME case reports in Kansas where this and other tick-borne diseases are a growing concern. We used annual county-level prevalence data for the period 2005–2012 and modeled the spatio-temporal autocorrelation and effects of independent covariates using Bayesian hierarchical modeling approach.

## Materials and Methods

### Data

#### Ethics statement

No patient names were recorded to maintain patient confidentiality and to adhere to the International Ethical Guidelines for Biomedical Research Involving Human Subjects. All patient records/information was anonymized and de-identified prior to analysis. The use of HME data was approved by the Internal Review Boards at Kansas State University and Kansas Department of Health and Environment (IRB # 6733).

#### HME data

Records of Kansas residents whose HME diagnosis or HME-related laboratory results were reported to the Kansas Department of Health and Environment (KDHE) from 2005 through 2012 were used in the study. Records that were classified as confirmed, probable or suspect as indicated by the Council of State and Territorial Epidemiologists' (CSTE) case definition were considered to be cases [12]. Further detail on case definitions is provided in File S1.

Cases were associated with their county of residence, due to the difficulty in determining where each infection originated, and because this was not routinely assessed or recorded.

#### Covariates

The publicly available 2006 National Land Cover Dataset [13] for the study region was obtained from the United States Geological Survey (USGS) in a raster grid format. Land cover grids within each county were extracted from the raster dataset and the percentage area they occupy were estimated. A list of land cover variables evaluated in the study is present in Table 1. Among climate variables, the maximum normalized vegetation index (NDVI); minimum land surface temperature (LST); mean LST; diurnal temperature range (DTR) (the difference between daily maximum and minimum temperature averaged over a thirty day period); precipitation and humidity were extracted for each county in the study area. The LST and NDVI estimates were derived from MODIS (Moderate Resolution Imaging Spectroradiometer) imagery [14]. DTR, precipitation and relative humidity were derived from the Prediction of Worldwide Renewable Energy (POWER) web portal of the NASA Langley Research Center [15].

**Table 1:** Land cover types found in NLCD.

Land cover land use data	Land cover types
National Land Cover Dataset (source: MRLC (2011); years^1^: 1992–2001; resolution^2^: 30 m; spatial scale^3^: 1∶100,000).	Open water, developed—open space, developed—low intensity, developed—medium intensity, developed—high intensity, barren land, deciduous forest, evergreen forest, mixed forest, scrub/shrub, grassland/herbaceous, pasture/hay, cultivated crops, woody wetlands, emergent herbaceous wetland.

1 Years represent the time period during which satellite images of land cover were captured for creating the data set, including multiple images within a year.

2 Resolution indicates the fineness of ground data as captured by a satellite image, shorter resolution meaning higher clarity;

3 Spatial scale indicates the scale for which interpretations are appropriate.

U.S. Census 2010 data on population and housing were obtained from the National Historical Geographic Information System (NHGIS), a publicly available online resource for U.S. Census Bureau's historical and current population data [16]. Identical census attribute information for Kansas was gathered at the county level. Geographic boundary files for counties were also obtained from the NHGIS. From the tables, 20 housing and 23 population related variables (Table 2) were extracted for each county by spatial query and joined to the census shapefiles using the common GIS codes.

**Table 2:** Population and housing variables evaluated in the study.

Census category	Independent variables^a^
Housing	*Housing units* (total housing units), *Tenure* (owner occupied, renter occupied), *Tenure (Historic or Latino Householder)* (owner occupied, renter occupied), *Race of householder* (white alone, Black or African American alone, Asian alone), *Household size* (1-person, 2-person, 3-person, 4-person, 5-person, 6-person, or 7-more person household), *Year structure built* (Built 2005 or later, 2000 to 2004, 1990 to 1999, 1980 or earlier^b^). (20 variables).
Population	*Population* (total population), *Race* (White alone, Black or African American alone, Asian alone), *Household income in the past 12 months* (Less than $10,000, $10,000 to $14,999, and thirteen other variables that represented $49,999 incremental income thereof up to $199,999, and $200,000 or more), *Poverty status in the previous 12 months* (income in the past 12 months below poverty level, income in the past 12 months at or above poverty level). (23 variables).

Definitions of different census variables can be found from their source (NHGIS) website at: https://www.nhgis.org/.

a Observations for all the independent variables are counts, in continuous form, and recorded per areal unit (block group, tract or county). Items in italics are Census Table names, and items within parenthesis are independent variables.

b The variable 1980 or earlier was derived by summing all the number of houses built prior to 1980 originally available in five-year increments in census.

### Statistical analysis and model specification

Let 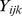 be the observed number of HME cases among 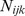 individuals at risk in the population of county 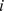, diagnosed with HME in year 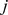 and of gender 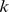. We modeled 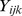 to follow a Poisson approximation, 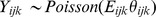, where 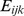 is the expected number of the population at risk for HME and 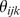 is the relative risk. Since HME prevalence is disproportionate among different age groups and gender [17], [18], standardized rates were calculated assuming 9 10-year age classes 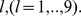 Thus, the expected number of HME cases was calculated by
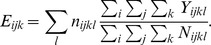



We used a logit link function in an extended generalized linear model (GLM) structure that incorporated stochastic spatial and temporal functions and as well as different covariate effects. Several models that allowed us to evaluate random and covariate effects on HME prevalence were fitted individually. First, a partial spatio-temporal model (partial ST) was fitted that was notated as following,
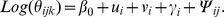



Where, 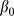 represents the mean prevalence of HME in all counties in all years, and 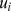 and 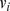 are random terms accounting for spatially structured variation in HME prevalence and unstructured heterogeneity, respectively. No interaction was assumed to exist between 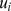 and 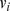 and were assigned 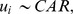 and 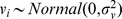 priors. Spatial dependence in
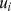
was applied by assuming a conditional autoregressive model 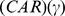 with a Gaussian distribution, which implies that each 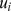 is conditional on the neighbor 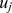 with variance 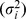 dependent on the number of neighboring counties 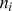 of county 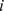, i.e.,
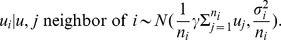



A random effect 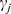 to account for the temporal component of the data was included and was assigned a random walk prior 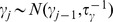 [19]. In order to detect potential spatio-temporal interaction effects in HME prevalence, 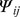 term was included in the partial ST model, which had a 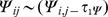 prior [20].

For the covariate model, different covariates were included to the partial ST model in several steps, starting with a model that included all covariates followed by removal of one variable at each step. Covariates were retained in the model unless their removal resulted in the increase of DIC value by 5 units or more. Several two-way interaction effects between covariates were also included in these steps. Candidate explanatory variables to be included in the Bayesian hierarchical models were screened *a priori* in order to avoid model fitting issues. Several frequentist bivariate logistic regression models evaluated each variable independently and only variables that were significant at *p*<0.2 were kept. A logistic regression takes the form,
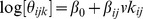



Where 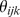 is HME relative risk, 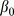 the intercept coefficient, and 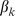 the coefficient for the explanatory variable 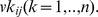 Care was taken not to remove candidate variables that were deemed clinically relevant [19]. Multicollinearity among screened variables was tested by estimating the variance inflation factor (VIF) and all variables with a VIF≥10 were considered to indicate multicollinearity [21], in which case, one of the variables was dropped at a time until multicollinearity was absent. Non-linearity among independent variables was evaluated at the screening stage with logistic regressions. Significant variables with non-linearity were categorized using cutoffs based on scatter-plots.

Model posterior parameters were estimated using a Bayesian framework implemented using R-INLA software [22] on a Linux Beocat cluster computing environment [23]. Distributions of covariate effects on HME prevalence are seldom available for the region; therefore non-informative, uniform priors were selected for the regression parameters, 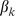 and their variance components, 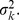 This allows the observed data to have the greatest influence on posterior distributions without being constrained by the choice of prior [24]. The mean estimates from the posterior distribution and their 95% credible intervals (CrI) were calculated and exponentiated to provide odds ratios (ORs) and their corresponding uncertainty measures.

Models were validated by randomly partitioning the county-level relative risk estimates into five subsets and by running the models using only four of the five subsets, while validating model prediction with the fifth subset. The models were run for five times to allow each validation with subset. Each time, the model's performance (prediction accuracy) was measured using area under the receiver-operator's curve (AUC) values with the observed prevalence (dichotomized as 0 or ≥0)”. The mean error and mean absolute error were calculated to quantify prediction bias and overall precision respectively.

## Results

There were 347 HME cases reported to KDHE between the years 2005–2012 predominantly distributed in the south and eastern counties of Kansas. A HME case in the dataset was under one of three categories: confirmed (n = 38), probable (n = 63), and suspect (n = 246). Table 3 shows a list of all candidate variables that were significant at *p<*0.2 level that were evaluated in this study, and Table 4 has the odds ratios and 95% CrIs for significant variables from the Bayesian spatio-temporal models. Non-linearity among significant variables was not observed. The best fitting model indicated significant spatio-temporal effect, income in the past 12 months below poverty level (henceforth, poverty), relative humidity, and an interaction term, ‘DTR x % mixed forest vegetation’ to be significantly associated with HME.

**Table 3:** Results of univariate logistic regression analysis of candidate covariates evaluated in the study.

Covariate	Odds ratio (95% CI)	*P-value*
Income in the past 12 months below poverty level	2.45 (2.02, 2.99)	0.04
Household size (>5 person)	1.61 (1.09, 2.39)	0.17
Relative humidity	2.77 (2.27, 3.37)	0.01
Minimum land surface temperature	1.34 (1.02, 1.77)	0.12
Diurnal temperature range	2.74 (1.03, 7.31)	0.17
% Mixed forest area	1.49 (1.00, 2.20)	0.18
Diurnal temperature range x % mixed forest area	1.82 (1.49, 2.21)	0.04

**Table 4:** Odds ratios and 95% Credible Intervals (CrI) from two spatio-temporal models evaluating county-level Human Monocytic Ehrlichiosis (HME) prevalence data in Kansas, USA.

Covariate	Partial ST model [Odds ratio (95% CrI)]	Covariate model [Odds ratio (95% CrI)]
Income in the past 12 months below poverty level	1.82 (1.49, 2.21)	2.22 (1.82, 2.70)
Relative humidity	3.38 (2.73, 4.20)	3.49 (2.81, 4.33)
Diurnal temperature range x % mixed forest area	3.00 (1.37, 6.57)	3.25 (1.48, 7.12)

Deviance information criterion (DIC) obtained by fitting a Bayesian equivalent was recorded as 3,754, and 2,472, and the σ^2^ (variance component) was 3.41 (1.31–4.81), and 2.33 (1.14–3.42) for the partial ST model and covariate models, respectively.

**Figure 1:**
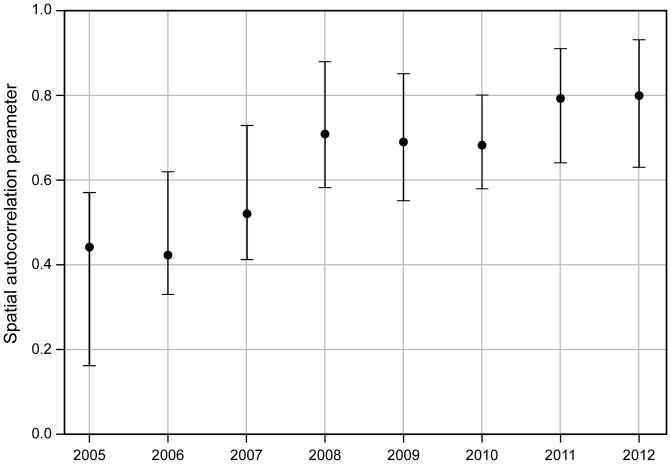
Spatial autocorrelation parameter (ψ_ij_) and 95% CrI for county level human monocytic ehrlichiosis (HME) relative risk between years 2005–2012 in Kansas.

**Figure 2:**
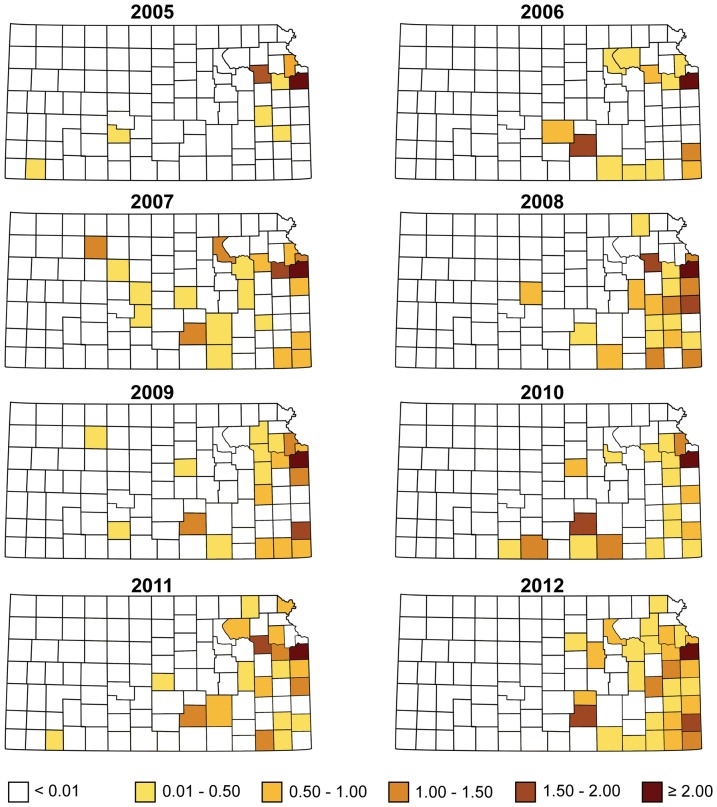
Kansas county level crude rate ratios for human monocytic ehrlichiosis (HME) in relation to total population (normalized by 10,000) between years 2005–2012.

**Figure 3:**
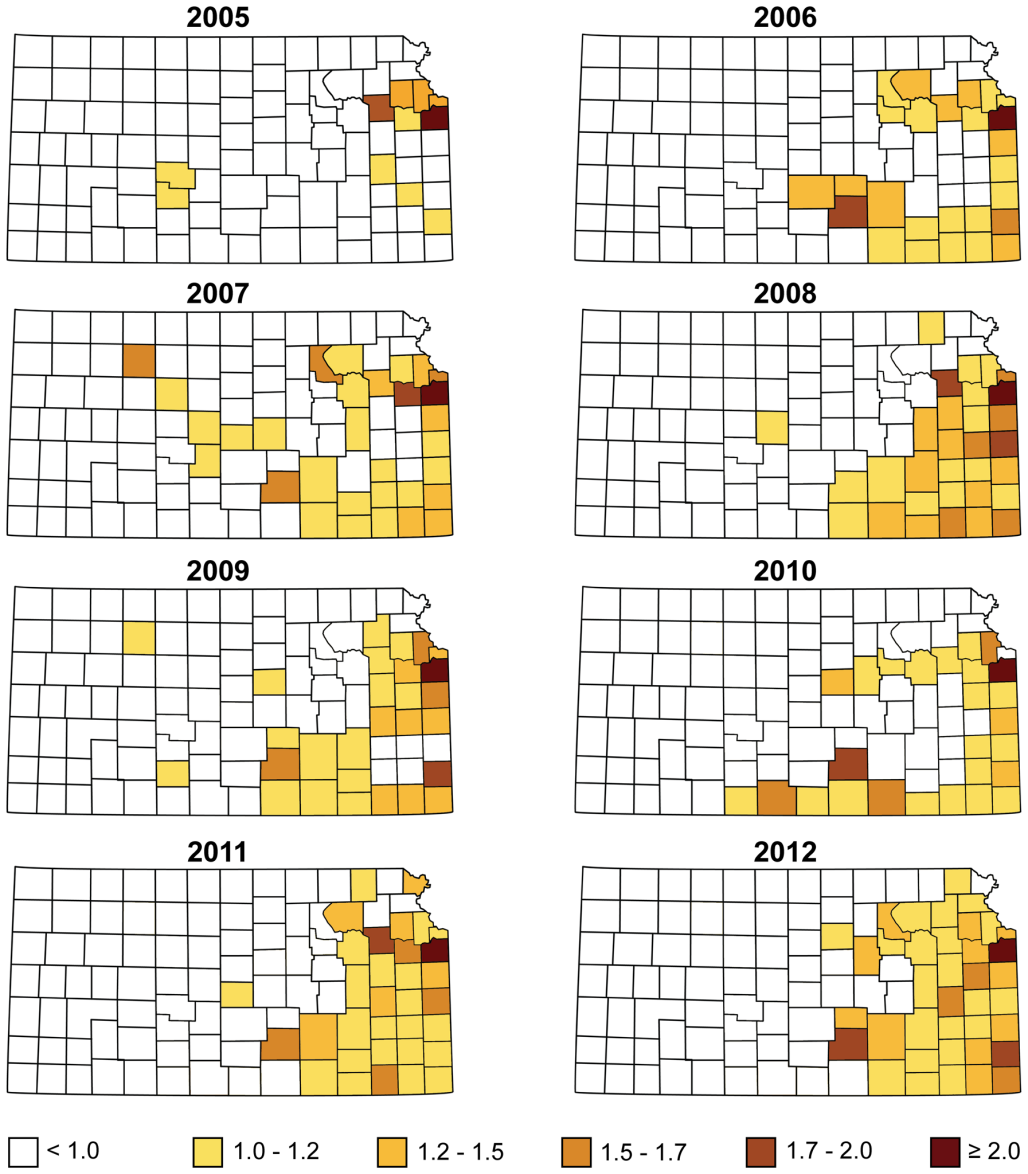
Smoothed relative risk maps for human monocytic ehrlichiosis (HME) in Kansas from 2005–2012.

The covariate model which incorporated terms for individual covariates in addition spatio-temporal interaction effect performed relatively better than the partial ST model for all years (Table 5), and all further interpretations were based on this model alone. The spatio-temporal autocorrelation parameter 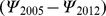 and their 95% CrI estimates quantified the infection risk between the counties over the study period and are plotted in Figure 1. The plot reveals positive and increasing autocorrelation between counties every year with a slight decrease in 2010–2011 period and only moderate increase during the latter part of the study period. The crude rate ratio of reported HME infections per county standardized by county population, and a smoothed map of posterior relative risk of counties between years 2005–2012 is shown in Figs. 2, 3, respectively. The posterior relative risk estimates are based on the final model with environmental, climatic and socio-economic predictors and correspond to the median of the posterior predictive distribution.

**Table 5:** Model validation summary for HME relative risk, 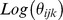
in Kansas between years 2005–2012.

Year	Model	AUC*	Mean error†	Mean absolute error (%)‡
2005	Partial ST	0.66 (0.61–0.71)	−0.14	6.12
	Covariate	0.69 (0.65–0.72)	−0.16	5.08
2006	Partial ST	0.66 (0.62–0.71)	0.11	6.27
	Covariate	0.72 (0.68–0.74)	0.09	5.14
2007	Partial ST	0.69 (0.59–0.72)	0.12	3.94
	Covariate	0.72 (0.69–0.74)	0.11	4.01
2008	Partial ST	0.72 (0.65–0.73)	0.14	5.21
	Covariate	0.74 (0.68–0.77)	0.12	4.88
2009	Partial ST	0.71 (0.58–0.72)	0.12	6.21
	Covariate	0.73 (0.70–0.75)	0.11	5.47
2010	Partial ST	0.69 (0.61–0.72)	0.10	4.22
	Covariate	0.76 (0.71–0.80)	0.09	4.18
2011	Partial ST	0.70 (0.65–0.71)	0.11	3.28
	Covariate	0.74 (0.68–0.75)	0.11	3.08
2012	Partial ST	0.71 (0.67–0.72)	0.08	4.88
	Covariate	0.73 (0.71–0.74)	0.09	3.97

*AUC values in the range of 0.5–0.7 indicates poor discriminative capacity, 0.7–0.9 is considered reasonable and >0.9 to be very good.

† Overall tendency to over or under-predict relative risk.

‡ Overall precision of models estimated using magnitude of error in predictions.

## Discussion

Results from this study shows that between years 2005–2012 there has been a general northwestwardly progression of HME prevalence in Kansas (Fig. 1), and a combination of climatic, environmental and socio-economic factors are important determinants of HME. It is notable that our results are potentially affected by our assumption that infections occurred within the county of residence even though the disease may have been contracted elsewhere. Only a fraction of cases were interviewed to determine where their infections originated, and their responses are subject to recall bias, another potential source of error. Another limitation in the study is the likelihood for clinicians in southeast Kansas to order more tests for HME than others where tick related illnesses are less of a concern. These limitations are typical for many retrospective spatial epidemiological studies, which can only be mitigated by conducting carefully designed prospective studies. Our study however underscores the need for such efforts in Kansas and the general region.

The overall spatial distribution of high-risk counties found in this study conforms to the approximate *A. americanum* tick distribution in Kansas, the primary vector for this disease, currently estimated by the CDC [25]. Although, CDC estimate of *A. americanum* distribution in Kansas could be an underestimate since their predictions were based on an acarologic survey conducted around the year 1945 [26]. It is likely for environmental and anthropogenic factors to have altered distribution of this species over these decades. The spatial pattern found in the present study is different from Wimberley et al., (2008) [4], which were mostly discontinuous and sparse within Kansas. This study considered a much larger spatial extent and had used deer serology results for prevalence data. Among all the geospatial variables evaluated in the present study, relative humidity, poverty status, and the combined effect of diurnal temperature range and mixed forest area appear to have played an important role in the spatial and temporal aspects of HME prevalence and in the vector/pathogen transmission cycle. While previous studies have evaluated various geospatial factors as determinants for ehrlichia ‘endemnicity’ [3]–[5], to our knowledge, this is the first time socio-economic factors were included and later identified as risk factors for HME using human incidence rates, and our Bayesian models provide some fresh information on HME spatial epidemiology in Kansas that are perhaps applicable to the broader region of the USA. This hypothesis, however, remains to be tested by conducting similar studies spanning various geographical regions of the USA. The current study lays the foundation for initiating such studies.

Humidity, the amount of water present in the atmosphere has been associated with tick survival in North America [27]–[29] and it is considered to be an important climate related delimiter to the spatial distribution of ticks [30]. There are large variations in the yearly precipitation received across the state of Kansas, with eastern Kansas receiving up to three times more rainfall than west [31]. As a result, climate and vegetation are transitional between the humid east and semi-arid western portion of Kansas that may explain the noted geographic pattern for HME in the present study. Humidity can often be seen associated with the survival and abundance of ticks in the literature, with higher humidity conditions often favoring the long-term survival of some ticks species' life stages through dry seasons [32], [33] among other reasons. Also, higher humidity conditions that are typically recorded during late spring and summer months correlate with higher human outdoor activities, which may increase exposure to infected ticks.

The interaction effect between diurnal temperature range and % mixed forest area towards HME prevalence is similar to our previous finding of the same association with feline cytauxzoonosis in the region [9]. Like HME, this disease is also transmitted by *A. americanum* ticks, but to felids and several wildlife hosts. One interpretation of the noted interaction effect could be that it is indicative of ticks' biological response to changes in DTR, but that is only specific to mixed forest areas and no other land cover types. Although previous studies have found that the host-seeking behavior of ticks [34] and the survival of tick-borne parasites [35] to be strongly influenced by DTR, the reason for interactive effect with mixed forest area is not clearly known. Mixed forest areas in the study region are likely suitable deer habitats, and are defined by the EPA as areas dominated by trees generally greater than 5 meters tall, and greater than 20% of total vegetation cover. Neither deciduous nor evergreen species are greater than 75 percent of total tree cover in mixed forests [13]. We suspect that in addition to changes in DTR, factors such as deer density, *E. chaffeensis* prevalence among deer and mixed forest wildlife hosts, and human interactions with mixed forest areas for recreation and hunting activities could be reasons behind this finding. *A. americanum* is an important vector for many pathogens in the region, and they appear to be influenced by DTR based on our previous and present studies. Laboratory examinations of DTR effects on the phenology and host-seeking behavior of *A. americanum* and the pathogenicity of ehrlichial pathogens the ticks carry are warranted.

The mechanistic basis for humidity-HME linkage and DTR-mixed forest interactive association with HME at the organismal level (for both *A. americanum* and *E. chaffeensis*) is likely to involve multiple pathways and their understanding is important in the context of ecology and evolution of HME, and also in the context of climate change effects on vector-borne diseases. Schwartz (1995) [36] documented an increase in more humid air masses in the later part of the 20^th^ century for eastern Kansas and Missouri and attributed this increase to climate change. The number of reported HME cases in Kansas has increased during the 2005–2012 period, and expanded from extreme southeastern Kansas to a large area of eastern Kansas. This spatio-temporal expansion and changing climate could be related and the linkages is worthy of investigation. Similar suggestions for other tick-borne diseases can be found in the literature [37]–[39]. Also, DTR is considered to be an important climate-change index [40], [41] which has been steadily decreasing since the 1950s due to daily temperature changes [40], [42]. Identifying consistent associations of relative humidity and DTR (either directly or as an interactive factor) with tick-borne diseases from other geographic regions will be useful in our efforts to quantify climate change effects on tick-borne diseases.

Socio-economic status is not frequently associated with tick-borne diseases in the US. Recent studies in Europe however have found mainly poverty but also political and other socio-economic factors with tick-borne encephalitis (TBE) [43], [44]. Also, principal components analyses have revealed lower socio-economic conditions to be stronger predictors compared to climate related factors for the TBE outbreak in Europe during the past decade [45]. Although the diseases that are being discussed here are caused and transmitted by different species, the role of socio-economic factors on HME is worth considering in future investigations. It is noteworthy that in Kansas, the spatial pattern for HME found in the present study and the distribution of poorly ranked counties for health is remarkably similar with only rare exceptions [46]. Poverty association with HME could be related to individuals engaging in outdoor occupations and/or living in close proximity to areas that favor tick habitats. Poverty status could also be a proxy for weakened immune system among individuals, lower literacy levels, and less awareness towards tick-borne diseases and their prevention methods. Studies to quantify individual level socio-economic status on HME incidence and any disparities in access to health care among high risk counties would be useful in preventing this disease.

## Conclusions

This study has shown a steady spatio-temporal progression of HME prevalence in Kansas as indicated by the strong autocorrelation estimates and the smoothed maps, and has also found some previously unknown risk factors for this disease. HME incidence in Kansas and much likely other endemic regions in the USA are affected by a combination of factors including climate, land cover/land use and socio-economic conditions. Quantifying the linkages between meteorological factors and the distribution and phenology of *A. americanum* and *E. chaffeensis* is needed for both prevention of HME and also for understanding the ecology and evolution of this disease and climate-change impacts. Bayesian spatio-temporal modeling approaches have advantages over traditional Frequentist inference methods and hold promise for disease monitoring and evaluation purposes.

## Supporting Information

File S1 HME case selection criteria.
(DOCX)
