# Supplementary material for: Bayesian Spatio-Temporal Analysis and Geospatial Risk Factors of Human Monocytic Ehrlichiosis
Source: PLoS One. 2014 Jul 3;9(7):e100850. doi: 10.1371/journal.pone.0100850 (PMC4081574; doi:10.1371/journal.pone.0100850)
Supplement: File S1 — HME case selection criteria. (DOCX) [file pone.0100850.s001.docx]

From 2005–2007, a confirmed case was defined as individual with clinical evidence (acute onset of fever, headache, myalgia, and/or malaise) and laboratory evidence, including serological evidence of a fourfold change in Immunoglobulin G-specific antibody titer to *E. chaffeensis* antigen by indirect immunofluorescence assay (IFA) between paired serum samples, a positive polymerase chain reaction (PCR) assay and confirmation of *E. chaffeensis* DNA, immunostaining of ehrlichial antigen in a biopsy or autopsy sample, culture of *E. chaffeensis* from a clinical specimen, or identification of morulae in leukocytes with a positive IFA titer to *E. chaffenensis* infection. A probable case was defined as an individual with compatible clinical illness and either a single positive IFA titer or the visualization of morulae in leukocytes. Suspected cases were not defined by CSTE from 2007–2012, but defined by KDHE as individuals with some laboratory evidence of past or present *E. chaffeensis* infection, with no available clinical information (e.g., a laboratory report sent to KDHE). Since 2008, a confirmed case was defined by CSTE as an individual with clinical evidence (any reported fever and one or more of the following: headache, myalgia, anemia, leukopenia, thrombocytopenia, or any hepatic transaminase elevation) and laboratory evidence, including serological evidence of a fourfold change in Immunoglobulin G-specific antibody titer to *E. chaffeensis* antigen by indirect immunofluorescence assay between paired serum samples taken 2-4 weeks apart, detection of *E. chaffeensis* DNA in a clinical specimen, demonstration of ehrlichial antigen in a biopsy or autopsy sample by immunohistochemical methods, or isolation of *E. chaffeensis* from a clinical specimen in cell culture. A probable case was defined as an individual with compatible clinical criteria and supportive, but not confirmatory, laboratory evidence of *E. chaffeensis* infection. This may include a single (not paired) positive serological titer to *E. chaffeensis* antigen, or the identification of morulae in white cells by microscopic examination in the absence of other supportive laboratory results. Suspected cases were defined as individuals with some laboratory evidence of past or present *E. chaffeensis* infection, but no available clinical information (e.g., a laboratory report sent to KDHE). Comprehensive investigation of HME laboratory reports did not occur prior to 2012; unless the reporting laboratory or physician included clinical information alongside a laboratory report, this information was usually absent from the patient’s record, resulting in a suspect case classification. Cases that did not meet the above case definitions were classified as “not a case” (n = 8).
